# Focused Epicranial Brain Stimulation by Spatial Sculpting of Pulsed Electric Fields Using High Density Electrode Arrays

**DOI:** 10.1002/advs.202207251

**Published:** 2023-04-28

**Authors:** Vishal Jain, Mats Forssell, Derya Z. Tansel, Chaitanya Goswami, Gary K. Fedder, Pulkit Grover, Maysamreza Chamanzar

**Affiliations:** ^1^ Department of Electrical and Computer Engineering Carnegie Mellon University Pittsburgh PA‐15213 USA; ^2^ Neuroscience Insttitute Carnegie Mellon University Pittsburgh PA 15213 USA; ^3^ Department of Biomedical Engineering Carnegie Mellon University Pittsburgh PA 15213 USA

**Keywords:** epicranial, motor cortex, motor evoked potential, pulsed electrical stimulation

## Abstract

Transcranial electrical neuromodulation of the central nervous system is used as a non‐invasive method to induce neural and behavioral responses, yet targeted non‐invasive electrical stimulation of the brain with high spatial resolution remains elusive. This work demonstrates a focused, steerable, high‐density epicranial current stimulation (HD‐ECS) approach to evoke neural activity. Custom‐designed high‐density (HD) flexible surface electrode arrays are employed to apply high‐resolution pulsed electric currents through skull to achieve localized stimulation of the intact mouse brain. The stimulation pattern is steered in real time without physical movement of the electrodes. Steerability and focality are validated at the behavioral, physiological, and cellular levels using motor evoked potentials (MEPs), intracortical recording, and c‐*fos* immunostaining. Whisker movement is also demonstrated to further corroborate the selectivity and steerability. Safety characterization confirmed no significant tissue damage following repetitive stimulation. This method can be used to design novel therapeutics and implement next‐generation brain interfaces.

## Graphical abstract

Epicranial electrical stimulation with high spatial resolution evokes selective behavioral response using a high‐density array. High density epicranial current stimulation also allows steering of the electric field within the brain and can perform simultaneous multisite stimulation.

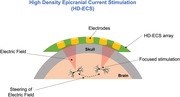

## 1 Introduction

Transcranial electrical stimulation (TES) is a powerful technique for brain interfacing, with numerous applications in the clinical realm^[^ ^1^, ^2^, ^3^, ^4^, ^5^ ^]^ as well as basic neuroscience research.^[^ ^5^, ^6^, ^7^ ^]^ Invasive neuromodulation modalities require complex surgical procedures and risk damage or infection to the brain tissue, making non‐invasive alternatives more appealing, especially if their spatial resolution can be improved. Some non‐invasive stimulation methods, like transcranial direct current stimulation (tDCS), transcranial alternating current stimulation (tACS), and focused ultrasound stimulation (FUS), are shown to affect neuronal excitability but are thought not to directly stimulate neurons by inducing time‐locked action potentials.^[^ ^8^, ^9^, ^10^, ^11^, ^12^, ^13^ ^]^ As such, these methods are of limited use in many research investigations and clinical applications that require measurable evoked activity.

Other non‐invasive stimulation methods, such as transcranial magnetic stimulation (TMS) and TES using high intensity current pulses have been shown to directly evoke action potentials.^[^ ^14^, ^15^, ^16^ ^]^ Existing TES and TMS used for human neurostimulation commonly rely on large electrodes and coils, respectively, and can generate a measurable output in muscles in the form of MEPs.^[^ ^17^, ^18^ ^]^ The use of large devices results in a large region of activation in the brain, which limits the target's specificity.^[^ ^19^, ^20^ ^]^ For many applications, the ability to target the stimulation to a specific region is of profound interest in order to avoid potential side effects or confounds due to off‐target stimulation.^[^ ^21^ ^]^

In addition to localized targeting, a desired feature is the ability to alter the location of stimulation after placing electrodes (i.e, electronic steering), which simplifies the electrode placement procedure by allowing for post‐setup calibration to take inter‐individual variation in target location into account. In addition, fast electronic steering allows for the stimulation of multiple locations simultaneously or sequentially, or of switching targets on the fly.^[^ ^22^ ^]^ This has recently been demonstrated using TMS^[^ ^7^, ^23^ ^]^ but not pulsed TES, although intersectional pulsed TES was used at a low intensity to improve the focus and targeting of transcranial electrical stimulation.^[^ ^7^ ^]^

While simulations of electric fields in human head models suggest that confinement of injected fields can be improved with HD arrangements,^[^ ^22^ ^]^ experimental validation of the direct effects of HD‐TES on neural responses is lacking. A recent work has used stereo EEG electrodes in human heads to measure the electric field in the brain during stimulation using low‐density electrode arrangements (for tDCS applications).^[^ ^24^ ^]^ Another recent work used concentric ring electrodes on rat skulls, a technique called epicranial current stimulation (ECS), to demonstrate that ECS applied through a focal electrode arrangement can result in a selective electromyography (EMG) response, measured as MEPs in forelimbs.^[^ ^25^ ^]^ Due to their geometry, the aforementioned concentric ring electrodes did not allow for steering of the focus, and physical movements of electrodes on the skull were used to demonstrate selectivity.

In this paper, we use epicranial HD electrode arrays (placed on a mouse skull) to generate a localized, tunable, and steerable electric field in the brain and evoke neural activity using short pulses of current injected through selected electrode configurations. The effect of stimulation is verified through three complementary methods: a) Selective stimulation of a forelimb detected by measuring MEPs and evoking whisker movements validated through video recordings, b) direct intracortical recording from the brain using multichannel penetrating neural probes, and c) molecular markers for neuronal activation using c‐*fos* immunostaining. We show that using an HD multielectrode array, multiple distinct stimulation outcomes can be achieved by steering the electric field with carefully chosen currents flowing through the electrodes. In addition to affecting the stimulation location, changes in the electrode configuration also affect the shape of the electric field in the brain, resulting in wider or narrower regions of stimulation.

To demonstrate this, we show in Section 2.1 that epicranial stimulation using HD arrays can successfully evoke a variety of selective behavioral responses, namely MEPs and whisker movements, and we confirm the neuronal origin of these effects using cellular markers for neuronal activity (c‐*fos*). In Section 2.2, electronic control of the electric field via HD‐ECS is further demonstrated by changing the selection of active and return electrodes to alter the electric field focus and location. This provides several degrees of freedom for electronically modulating neural activity by selecting electrode configurations. In Section 2.3, direct evidence of neural stimulation and focality from HD‐ECS is further shown by measuring evoked intracortical neuronal responses. Moving the stimulation focus allows us to quantify the extent of neural activation in the brain, confirming that stimulation is occurring on a very focal level. In Section 2.4, both steerability and focality are additionally confirmed at the cellular level using c‐*fos* immunostaining. Finally, in Section 2.5, we demonstrate the safety of HD‐ECS through a rigorous immunohistochemistry analysis using cellular and neurodegeneration markers.

## 2 Results

### 2.1 HD‐ECS Evokes Selective Behavioral Response

We first demonstrated the potential of HD‐ECS to evoke a behavioral response by stimulating the motor cortex through the skull in an in vivo rodent model. The use of a H‐D electrode array results in a rich design space to achieve distinct stimulation patterns by selecting the electric current amplitudes applied through individual electrodes. By using a subset of electrodes to carry current, distinct spatial patterns can be attained. Likewise, we illustrated that by simultaneously scaling the current amplitude at all electrodes, a distinct behavioral response can be elicited by using the same subset of electrodes. Specifically, whisker movements can be evoked at a lower current intensity, whereas MEPs in limbs are evoked at a higher current intensity. This also provides initial evidence that the response is focused and selective, as contralateral responses were dominant in both cases (MEPs and whisker movement).

**Figure 1:**
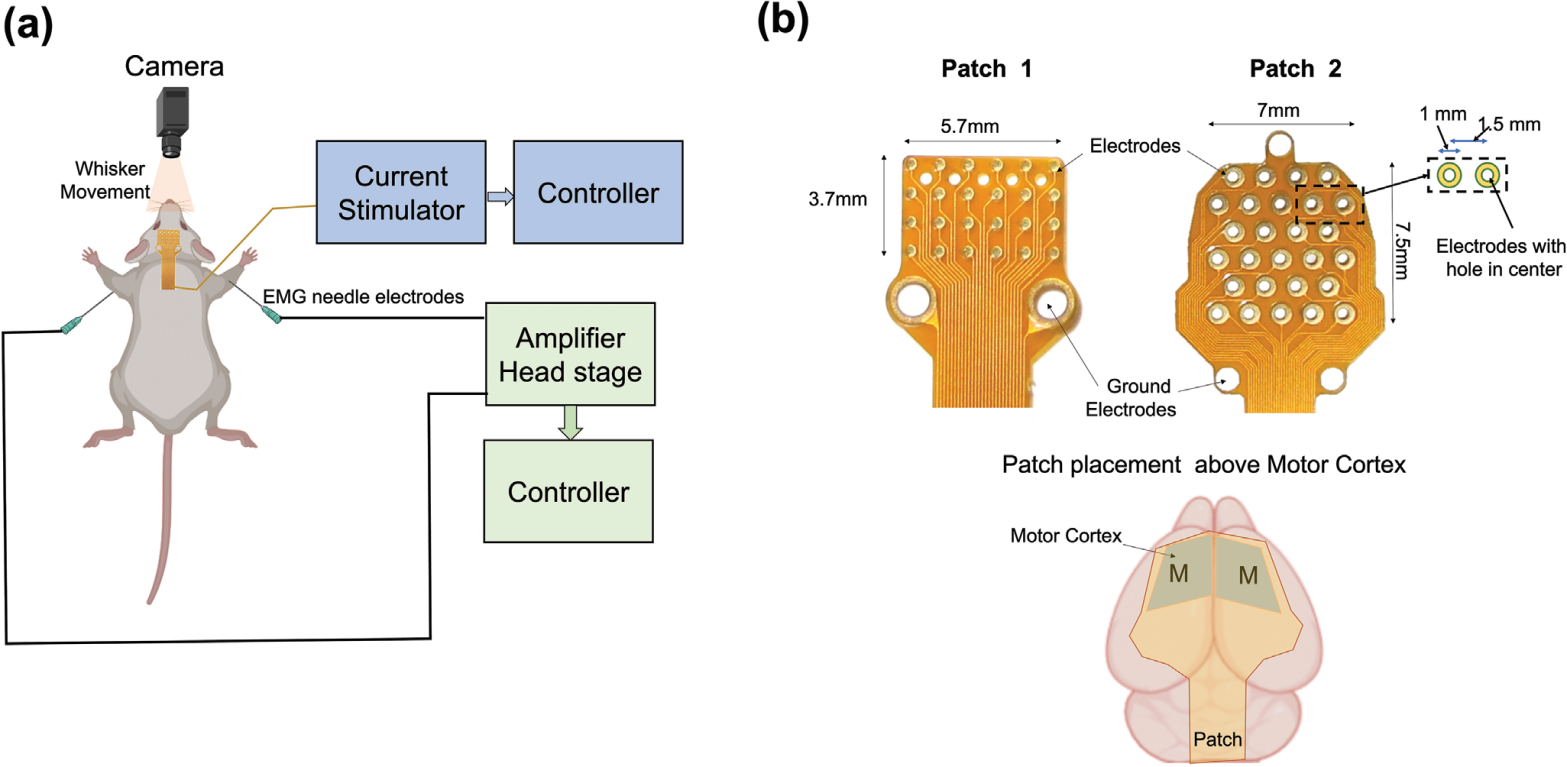
Experimental paradigm. a) Schematic representation of stimulation paradigm showing connection of the stimulation patch and EMG electrodes placement along with camera for whisker movement recording. b) Two different flexible patches were used for HD‐ECS. Patch 1 contained 24 disk electrodes arranged in a 6×4 array in a cartesian grid, and Patch 2 had 27 ring‐shaped electrodes arranged in a hexagonal grid. The patch was placed above the skull with its center aligned to bregma, covering the motor cortex of both hemispheres.

To conduct in vivo experiments, a custom‐designed flexible surface electrode array was directly placed over the exposed mouse skull to stimulate the motor cortex (**Figure** **1**b). MEPs were recorded in both forelimbs using EMG electrodes. Additionally, whisker movements were captured using a video camera (Figure 1a). For this study, a flexible surface electrode array with 24 electrodes was designed in a 6 × 4 grid arrangement, as shown in Figure 1b.

**Figure 2:**
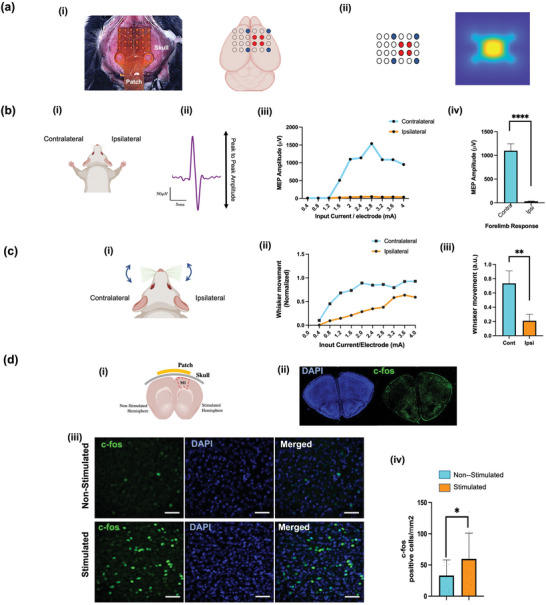
Validation of HD‐ECS mediated activity using different measurement modalities. a) Stimulation configuration: i) HD electrode patch on skull, targeting motor cortex of right hemisphere; 4 + 4 electrode stimulation pattern configuration is shown (red: active electrodes, blue: return electrodes). ii) Corresponding simulated electric field at depth of 0.8 mm in brain (corresponding to cortical layer L5). b) EMG results: i) EMG electrodes were inserted in both forelimbs. ii) Representative MEP response following HD‐ECS. iii) Amplitude of MEP at different input currents (average of 10 repetitions for each condition). iv) Comparison between contra‐ and ipsilateral amplitudes at 2 mA injected current (mean ± SD, *n* = 7 animals) showing significant difference (*p* = 0.0001, Welch's *t*‐test). c) Whisker results: i) Bilateral whisker movement was recorded following stimulation. ii) Average amplitude of whisker movement quantified at different currents (average of 10 repetitions for each condition). iii) Comparison between contra‐ and ipsilateral whisker movement amplitude at 1.2 mA injected current (mean ± SD, *n* = 6 animals) showing significant difference (*p* = 0.05, Welch's *t*‐test). d) Cellular‐level results: i) Stimulation was further validated by assessing neuronal activation using c‐*fos* immunostaining in both hemispheres: stimulated (right) and non‐stimulated (left) hemispheres. ii) Section below patch surface was selected for evaluation and stained with c‐*fos* and co‐labeled with DAPI. iii) Representative images from both groups with c‐*fos* and DAPI stains with their merged impression (scale bar: 50 µm). iv) Quantitative graphical representation showing significant difference between both hemispheres; significance was analyzed using Welch's *t*‐test, *p < 0.05.

This design provided bilateral electrode placement above the motor cortex and allowed us sufficient freedom to tailor the stimulation field to select targets with high resolution while keeping the electrode impedances low. To apply the stimulation, we used eight electrodes out of the total 24 available electrodes (**Figure** **2**a(i)): four active electrodes for injecting currents surrounded by four return electrodes, which collected electric current from the tissue back into the stimulation electronic circuitry. The injected current was evenly distributed across the four active and four return electrodes (Figure 2a(ii)). This 4 + 4 stimulation electrode arrangement in the square lattice was designed to uniformly cover the entire motor cortex of the right hemisphere and was thus expected to elicit MEP responses in the left (contralateral) forelimb (Figure 2b(i)). The response to stimulation was quantified by the peak‐to‐peak amplitude of the MEP signal measured through pairs of EMG electrodes implanted in both forelimbs (Figure 2b(ii)).^[^ ^26^ ^]^ An input‐output curve between input current and MEP amplitude was generated by gradually increasing the current intensity from 0.4 to 4.0 mA (total injected current) and used to determine the motor threshold (Figure 2b(iii)). Specifically, the motor threshold is defined as the current required to elicit an MEP amplitude of 50% of the maximum MEP amplitude measured.^[^ ^27^ ^]^ Results showed that the motor threshold for the contralateral limb was 2 mA. An ipsilateral response was also observed (Figure 2b(iii)), but it was not significant. At the motor threshold, the response in the contralateral forelimb was significantly higher (*p* < 0.0001) than in the ipsilateral forelimb (Figure 2b(iv)), which demonstrates the selective nature of HD‐ECS. Additionally, using the same 4 + 4 electrode arrangement, whisker movement was measured using video recordings. The amplitude of whisker movements on both sides was calculated. As for MEP characterization, an input‐output curve was established between injected current and whisker movement ranges, from 0.4 to 4 mA (Figure 2c(ii)). Using the same definition as the motor threshold, the threshold for whisker movement was achieved at 1.2 mA in contralateral whiskers; at this current, the contralateral movement was significantly higher (*p* < 0.01) than ipsilateral whisker movements. An ipsilateral response was observed, but the contralateral response was always more prominent. Similarly Carmel and Martin found a few projections from the motor cortex onto the ipsilateral half of the spinal cord, and intracortical microstimulation (ICMS) currents required to evoke an ipsilateral movement are 2.4 times larger than those required to produce a contralateral movement.^[^ ^28^ ^]^ It is also interesting to note that selectivity to different behavioral responses can be achieved by modulating current intensity; the current threshold to evoke whisker movement was lower compared to the current threshold for limb movements.

Further, we validated that HD‐ECS mediated stimulation in the target region(i.e., motor cortex) by studying neural activation following stimulation at the cellular level using c‐*fos* immunostaining, a marker of functional neuronal activation,^[^ ^29^ ^]^ in the stimulated and non‐stimulated hemispheres (Figure 2d(i)). For this validation study, a similar flexible electrode array was used, and a motor threshold was identified by applying a sweep of currents. After identifying the motor threshold (2 mA), 200 repetitions of stimulation were applied to establish the determined effect. Following the stimulation, a 60 minute recovery period was given to allow for persistent expression of neuronal activation markers. Coronal sections of the motor cortex (both stimulated and non‐stimulated regions) were used for studying c‐*fos* expression. Results revealed an increase in c‐*fos* activity in the stimulated hemisphere compared to the non‐stimulated hemisphere (Figure 2d(iii)), as evidenced by the significantly higher (*p* < 0.01) number of c‐*fos* positive cells in the stimulated hemisphere, with a mean difference of 26.83 ± 13.93 cells in a 3×3 mm field of view (Figure 2d(iv)). This confirms that activation of neurons in the stimulated hemisphere was responsible for the observed behavioral response, (i.e., MEPs) in the forelimbs. These findings highlight the ability of HD‐ECS to evoke selective contralateral movement. In addition to modulating the current amplitude, the HD electrode array allows for tuning of the injected electric field to achieve various stimulation outcomes.

### 2.2 HD‐ECS Enables Electronic Control of Stimulation Region

**Figure 3:**
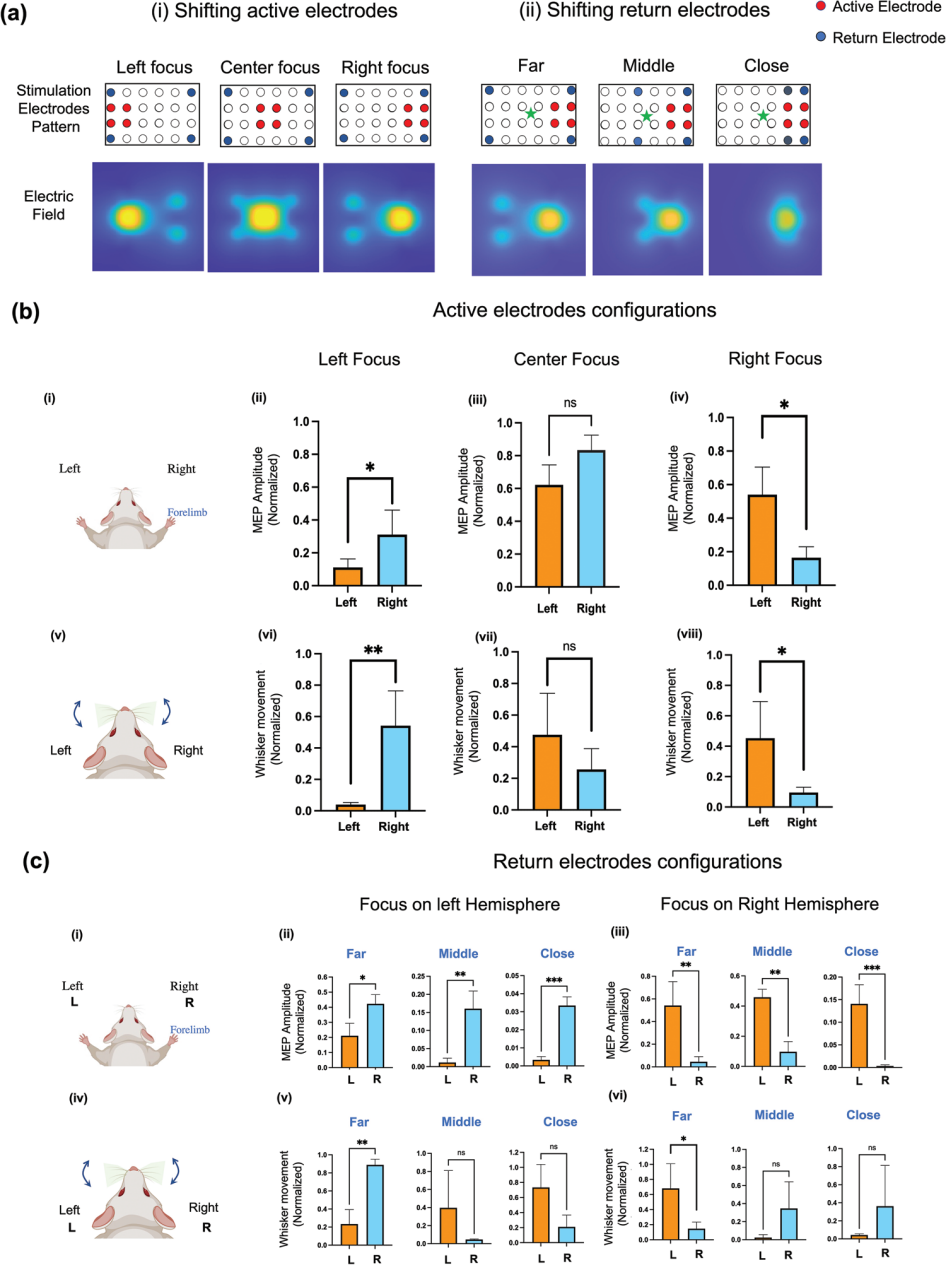
Control of stimulation location and extent with HD‐ECS. a) Simulated electric field for different current‐injection configurations. Fields are plotted in a longitudinal plane at a 0.8 mm depth in the brain (corresponding to cortical layer L5). The corresponding current‐injection configuration is shown for each simulation; in each configuration, all active electrodes have the same injected current, and all return electrodes have the same injected current. i) Active electrodes are moved to target left, center, and right hemisphere of brain while positions of return electrodes are kept static. ii) Return electrodes are moved to form three different configurations— *far*, *middle*, and *close*—on each hemisphere. b) Movement of active electrodes. ii–iv) Normalized MEP amplitude, and vi–viii) Whisker movement corresponding to indicated configuration. The injected current used was 2 mA for MEPs and 1.2 mA for whisker movement. Data represent mean ± SD, (*n* = 5 for MEP, *n* = 4 for whisker movement). Significance calculated using Welch's *t*‐test *p < 0.05, **p < 0.01. ns = non‐significant. c) Movement of return electrodes. ii,iii): Normalized MEP amplitude, and v,vi): Whisker movement for indicated configuration. The injected current was 2 mA for MEPs and 1.2 mA for whisker movement. Data represent mean ± SD, (*n* = 5 for MEP, *n* = 4 for whisker movement). Significance calculated using Welch's *t*‐test *p < 0.05, **p < 0.01. ns = non‐significant.

We demonstrated HD‐ECS' ability toflexibly steer fields across different regions of the brain through the skull by electronically controlling the currents injected through different electrodes. Arbitrary selection of the electrodes through which current flows (i.e., active electrodes) enables the designing of different patterns of stimulation without physically moving electrodes. Different degrees of steerability can be achieved by changing the active electrode arrangements. The subset of active electrodes can be translated on the electrode grid without changing their relative position, emulating physical movement of the pattern on the head to target different regions of the brain. In addition to translation of the same field pattern, the stimulation pattern can be changed to elicit a different electric field shape by recruiting different electrode arrangements. In this study, we experimentally demonstrate two different approaches to steer currents within the brain: (i) Moving active electrodes—translating the active electrodes on the grid while keeping the return electrodes stationary, which shifts the location of the electric field maximum (**Figure** **3**a(i)); and (ii) Moving return electrodes—changing the return electrodes on the grid while keeping the active electrodes stationary, which changes the confinement of the electric field (Figure 3a(ii)). We report the experimental results on three adult mice.

#### 2.2.1 Control of Stimulation Location by Shifting Active Electrodes

The flex‐PCB with a 24‐electrode array in a 6 × 4 grid arrangement was placed and centered on the mice skull's bregma. We used variations of the 4 + 4 electrode arrangement (Figure 3a(i)) and assessed the effect of steerability by measuring the amplitudes of MEPs and whisker movement. For each configuration, a sweep of current intensities ranging from 0.4 to 4 mA were applied to determine the motor threshold, which was determined to be 1.6 mA. For each limb, the MEP amplitude was normalized to the maximum response measured across all currents and patterns, corresponding to the maximum possible activation of that limb. The stimulation pattern with active electrodes in the center evoked an almost identical MEP response in both forelimbs (Figure 3b), substantiating the HD‐ECS‐mediated stimulation beneath the active region because, in this case, the active region covered the motor cortex of both hemispheres. Using the same injected current intensity, when the set of active electrodes was translated to the left by 2 mm, a selective contralateral response in the right forelimb (0.3 ± 0.14, normalized MEP amplitude) was observed, which had a significantly (*p* < 0.01) higher MEP amplitude than the left forelimb (0.11 ± 0.05, normalized MEP amplitude) (Figure 3b(iii)). Similar results were found when the active electrodes were translated to the right side of bregma by 2 mm, as evidenced by a significantly (*p* < 0.01) higher MEP response in the left forelimb (0.54 ± 0.16, normalized MEP amplitude) compared to the right forelimb (0.16 ± 0.06, normalized MEP amplitude) (Figure 3b(iii)). At higher currents, some ipsilateral response was observed, but the contralateral response was more prominent.

In addition to limb response, whisker movements evoked by the same patterns of stimulation were also studied. As for the forelimbs, the center‐focused pattern evoked bilateral whisker movement with no significant difference between the left and right whiskers, while the pattern focused on the left and right sides elicited a distinct contralateral response in the right (*p* < 0.01) and left (*p* < 0.05) whiskers, respectively (Figure 3b(iv)). The injected current amplitude was swept from small values to higher values to determine the threshold required to stimulate whisker movement, which was found to be 1.2 mA. This threshold was lower than the threshold required to evoke a motor response. Additionally, whisker movement seemed more spatially selective than the limb response, as no statistically significant ipsilateral whisker movement was observed even at high current amplitudes.

#### 2.2.2 Control of Stimulation Focus by Shifting Return Electrodes

The effect of moving return electrodes was also studied using the same 6 × 4 electrode grid array, identically placed on the mouse skull centered on bregma. We expected that moving the return electrodes closer to the active electrodes would enhance the confinement of the electric field in the brain and hence improve the stimulation focus, resulting in a more selective response. To demonstrate this effect, the 4 + 4 electrode arrangement was used in three different configurations(i.e., the *far*, *middle*, and *close* arrangements) based on the distance between the return electrodes and active electrodes (Figure 3a(ii)). The *far* configuration used return electrodes at the corners of the grid array, which maximized the distance to the active electrodes and was expected to create a wider spread of the injected currents in the tissue. In the *middle* configuration, the return electrodes were moved closer to the four active electrodes by 1 mm on average, and in the *close* configuration, the return electrodes were adjacent (as close as possible) to the active electrodes. Simulations showed the extent of the generated electric field in each configuration (Figure 3a(ii)) and confirmed that the electric field was more focused when the return electrodes were closer to the active electrodes. All three configurations were applied to both hemispheres independently. The MEP response in forelimbs and whisker movements were observed using the three different stimulation patterns. As anticipated, in the *far* arrangement, a distinct contralateral response with a motor threshold of 1.6 mA was observed (Figure 3c(iii)). With different return electrode arrangements, a persistent contralateral response was observed, but the motor threshold gradually increased from 1.6 mA, 2 mA, and 2.8 mA in the *far*, *middle*, and *close* configurations, respectively. Interestingly, the MEP amplitude decreased when the return electrodes moved in the *far*, *middle*, and *close* configurations (Figure 3c(iii)). This change in motor threshold and MEP response may be attributed to the increase in stimulation focus coupled with a decrease in electric field amplitude at a particular depth (Figure 3a(ii)). Overall, using the *close* arrangement, we can achieve a higher selectivity for contralateral stimulation of the limbs. We note, however, that this arrangement requires a higher injected current amplitude. Consistent with our findings in this paper, previous studies have also shown that in simulations, transcranial current injection with return electrodes closer to active electrodes increases focality and requires a higher current intensity to achieve stimulation.^[^ ^22^ ^]^

Whisker movement, however, follows a different trend using these same patterns. With *far* return electrodes, a large contralateral whisker movement was observed, associated with a small ipsilateral whisker movement. Unlike the forelimb response, when the return electrode was switched to the *close* configuration, an ipsilateral whisker movement was preferentially evoked. The amplitude of ipsilateral movement with the *close* returns was statistically identical to that obtained with the *far* returns. However, in the intermediate configuration of the *middle* returns, no significant whisker response was evoked. The presence of ipsilateral whisker movement but not ipsilateral MEP in the *close* configuration points to a difference in response to transcranial currents of the whisker and forelimb systems. Moreover, the lack of ipsilateral whisker movement in the *middle* configuration points to there being different pathways of ipsilateral whisker activation in the *far* and *close* cases. The additional ipsilateral activation may be attributed to the activation of some interhemispheric network connections responsible for whisker movement on the ipsilateral side.^[^ ^30^ ^]^ These findings enable the possibility of using several electrode arrangements, leading to improved confinement of the electric field to achieve focus stimulation with higher spatial resolution.

### 2.3 Validation of Focality Achieved by HD‐ECS Using Intracortical Neural Recordings

**Figure 4:**
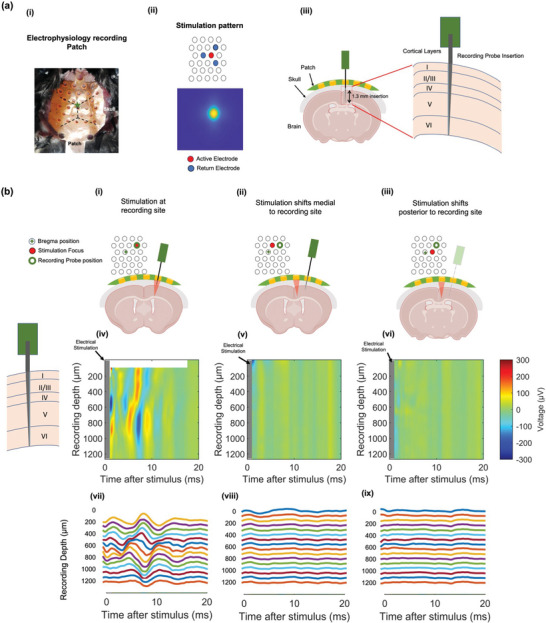
Intracortical activity during HD‐ECS demonstrates spatial focality. a) Setup of intracortical recording experiments. i) Patch2 placed on mouse skull targeting motor cortex. ii) 1×3 electrode configuration used for stimulation and longitudinal cross‐section of simulated electric field at 0.8 mm depth in brain. The same current amplitude is injected at all return electrodes. iii) Schematic of setup for simultaneous HD‐ECS and intracortical recording. A hole is drilled in the skull, through which the 64‐channel recording probe is inserted to cover the entire cortical column. b) Intracortical recordings (MUA) validating focality of HD‐ECS. i–iii) Schematic of stimulation setup showing coronal cross‐section of patch and brain at different locations. The recording probe is at the same location in all cases (AP = 1 mm, ML = 1.5 mm), and the stimulation current configuration (focus) is moved between three different locations. (i,iv): AP 1 mm, ML −1.5 mm, at the recording location; (ii,v): AP 1 mm, ML −0.5 mm, medial to recording location; and (iii,vi): AP 0 mm, ML −1 mm, posterior to recording location. iv–vi): Spatiotemporal profile of MUA, plotted as voltage across time throughout cortical laminae (1200 µm) following stimulation. Plots (vii–ix) represent the same data as (iv–vi), showing waveforms measured at the recording probe following stimulation at positions (i–iii), respectively (1 in 4 channels are shown for readability). The missing data at the top of plot (iv) are due to saturation of the recording amplifier.

In order to further validate the direct effect of HD‐ECS on the brain, we measured intracortical activity in the motor cortex resulting from the stimulation. Additionally, we utilized the multi‐electrode capabilities to steer the current in order to quantify the focality achieved in the brain. We optimized the electrode arrangement through simulations,^[^ ^31^ ^]^ allowing us to use smaller electrodes with reduced pitch without compromising our ability to inject high enough levels of current into the brain. To achieve this, a dedicated flexible PCB electrode array was designed, consisting of an array of ring electrodes (470 µm outer diameter) with 350 µm diameter holes at their center to allow insertion of the recording probe at the stimulation target location (**Figure** **4**a(i)). To allow closer packing of electrodes (and thus enable fine‐grained measurements), this patch was designed to have a hexagonal symmetry instead of the Cartesian grid used previously. A 1 + 3 electrode arrangement was used, in which one center active electrode is surrounded by three adjacent return electrodes with identical currents (Figure 4a(ii)). To avoid interference of the stimulation artifact with the electrophysiological response, only a single stimulation pulse was used. The evoked activity was recorded in the entire cortical column using a 64‐channel silicon neural recording probe (Figure 4a(iii)). To assess the focality of stimulation, the recording probe was inserted at one target location (AP 1.3 mm; ML −2.3 mm; from bregma) to record multi‐unit activity (MUA). The focus of stimulation was steered such that the theoretical electric field maximum was achieved at three different locations: the recording location (AP 1.3 mm; ML −2.3 mm, Figure 4b(i)), medial to the recording location (AP 1.3 mm; ML −0.8 mm, Figure 4b(ii)), and posterior to the recording location (AP 0 mm; ML −1.5 mm Figure 4b(iii)). The stimulation focus was steered to the three target locations by electronically moving the same injected current pattern. This was achieved by selecting different subsets of electrodes to induce the same electric field translated to different locations (Figure 4b). Results showed that HD‐ECS evoked neuronal activity when the stimulating electric field was centered at the targeted location, as evidenced by MUA throughout the cortical column. The MUA propagated along the column within 20 ms due to cortical network connections (Figure 4b(iv)). When the center of the injected current was moved either medially (Figure 4b(v)) or posteriorly (Figure 4b(vi)), no MUA was observed. This shows that with the current‐injection pattern used here, the extent of HD‐ECS‐mediated stimulation is confined within a 1.5 mm range around the stimulation center and substantiates the ability of HD‐ECS to evoke a focused response.

### 2.4 Cellular‐Level Validation of Spatial Focality and Steerability of HD‐ECS

**Figure 5:**
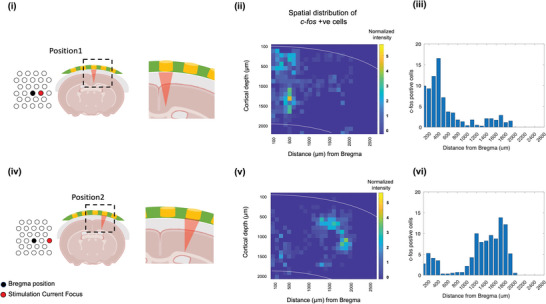
Validation of HD‐ECS steerability and focality at cellular level. Cellular validation of focused and steerable neural activity following HD‐ECS. The same 1 × 3 electrode arrangement was used for stimulation as in the MUA recordings (Figure 4a(ii)). Stimulation current was injected to target two different positions. i–iii) Immunostaining results from stimulation using configuration in Figure 4a with center at AP 0 mm, ML −0.5 mm (Position 1). iv–vi) Stimulation with center at AP 0 mm, ML −1.5 mm. i,iv): Experiment schematic showing stimulation center represented on patch (left), coronal section of patch and brain around the stimulation location (center, right). ii,v) Map of density of c‐*fos* positive cells (cell counts in 100 µm × 100 µm blocks). White lines represent the edge of the cortex. iii,vi) Cell count of c‐*fos* positive cells integrated along the cortical depth.

We also confirmed HD‐ECS' steerability and focality at the cellular level by studying neuronal activation using the immediate early gene c‐*fos* as a marker of functional neural activation.^[^ ^29^ ^]^ The c‐*fos* gene has been widely used as an indicator of activated neurons in mice and rats,^[^ ^24^ ^]^; therefore, it can be used to identify the stimulated region in the brain to further confirm the focality and steerability following stimulation. In the current study, we utilized c‐*fos* as a marker to validate the steerability by studying the change in c‐*fos* expression in the motor cortex following HD‐ECS. Studying c‐*fos* expression as a neural activity marker provided information about the extent of stimulation at the cellular level and also validated the steering based on the change in location of c‐*fos* positive cell clusters after applying stimulation at two different locations. For this experiment, the same electrode arrangement, but with a different stimulation focus, was applied in two animals to elicit a response at two adjacent locations (**Figure** **5**(i),(iv)). We used the same 1 + 3 electrode arrangement discussed in the previous section (Figure 4a(ii)), but the injected current waveform was changed to a 7‐pulse train to induce a motor response. The motor threshold was adopted from previous experiments, (i.e., 2 mA and 200 repetitive stimulations were applied at a 0.5 Hz interval). Following stimulation, animals were sacrificed after 60 min to allow persistent expression of c‐*fos* following stimulation. To quantify the spatial distribution of c‐*fos* expression, c‐*fos* positive cells were counted and binned within 100 × 100 µm on a 1.6 mm × 2 mm image covering the motor cortex in the stimulated hemisphere. Results were normalized with c‐*fos* expression in control sections, without any stimulation to avoid intrusion due to baseline c‐*fos* activity. The result showed a distinct spatial shift in neuronal activity, evidenced by shifting of the cluster of c‐*fos* positive cells in accordance with the position of focused currents (Figure 5(ii),(v)). As shown in Figure 5(iii), the peak of c‐*fos* positive cells lied in the range of 200–600 µm when stimulation focus was at position 1 (Figure 5(i)) and in the range of 1400–2000 µm when stimulation focus was moved by 1500 µm to position 2 (Figure 5(iv),(vi)). The center of the cluster of c‐*fos* positive cells shifted by 1200 µm between the two patterns, which is very close to the shift in the targeted stimulation focus (1500 µm). Notably, at both positions, the extent of the positive clusters covered a range of 400–600 µm, which further substantiates the focality of HD‐ECS.

### 2.5 Safety Characterization of HD‐ECS

**Figure 6:**
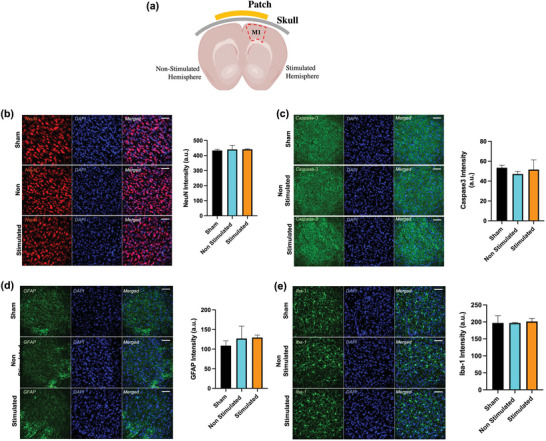
Safety assessment following HD‐ECS. For safety characterization, we assessed different cellular markers for cell type and damage in three different groups: sham, non‐stimulated, and stimulated. a) Schematic of experimental setup showing a coronal cross‐section of the brain with stimulation patch targeting motor cortex of one hemisphere. In the stimulated group, 200 stimulation trains were delivered at a rate of 0.5 Hz. :b) Following stimulation, neuronal density was studied using NeuN immunostaining co‐labeled with DAPI in different groups and analyzed with NeuN intensity. c) Cleaved caspase3 was used to study apoptosis and caspase3 intensity was compared between different groups. d) Gliosis was studied in microglia using GFAP staining. e) Gliosis was further evaluated in astrocytes using Iba‐1 as a marker of immune response against damage, and its intensity was compared between different groups. One‐way ANOVA was used for significance; three sections from each brain (*n* = 9) were taken. Scale bars represent 50 µm.

In addition to demonstrating the efficacy of HD‐ECS for focal and steerable stimulation of the brain's local circuits, its safety was assessed by studying the expression of cellular markers for cell death and cell types following HD‐ECS in one hemisphere. For histological experiments, the 4 + 4 electrode arrangement was used with active electrodes targeting the right hemisphere with the *right focus* configuration (Figure 3a). This configuration elicited a widespread electric field and hence evoked the largest motor response. A sweep of current was applied to determine the motor threshold, and then a train of seven pulses was applied with 200 repetitions at 0.5 Hz. Samples were collected 60 minutes after the end of stimulation, which permitted sustainable expression of cellular markers. To characterize the safety profile of HD‐ECS, different cellular markers were studied to assess neuronal density (NeuN), gliosis (Iba‐1 and GFAP), and apoptosis (cleaved caspase‐3) following stimulation. A comparison was made in the fluorescence intensity of the stimulated versus non‐stimulated hemispheres of the experimental group (Figure 6a), and with sham groups that underwent the same procedure without any stimulation. No significant changes were observed in neuronal density between the stimulated (441.2 ± 3.3) and non‐stimulated (440.6 ± 27.6) groups or with the sham (433.9 ± 8.1) group (Figure 6b). Similarly, no change in apoptosis was observed between the different groups, as evident from caspase‐3 expression (Figure 6c). This experiment shows that HD‐ECS causes no significant neuronal damage. In addition to these markers, gliosis was also studied by measuring the concentration of microglia (GFAP) and astrocytes (Iba‐1). High concentrations of these cells indicate a neuroimmune response of brain cells. HD‐ECS did not cause any gliosis, as no significant difference in intensity of glial (Figure 6d) cells or astrocytes (Figure 6e) was observed across different groups. This finding indicates a lack of reactive microglial or astrocytes in response to HD‐ECS. These observations provide sufficient evidence that the HD‐ECS approach does not cause any cellular damage, as no changes in neuronal density and no neuroinflammation markers were observed following repeated stimulations.

## 3 Discussion

We demonstrated that a high‐resolution, minimally invasive epicranial current injection approach using HD‐ECS can evoke localized neural activity as well as a downstream behavioral response with focalized stimulation in the brain. This technique uses a multi‐electrode array to enable steering of current within the tissue and can evoke neural and behavioral responses in different brain regions without physically moving the electrodes.

To achieve this goal, HD‐ECS was applied on one hemisphere of the mouse brain using a 4 + 4 electrode arrangement to cover the major motor cortex. Successful forelimb and whisker movement were observed. We further validated the effect of HD‐ECS on the brain at the functional level by measuring MUA and at the cellular level by measuring c‐*fos* expression in the motor cortex. The complementary data provided by the EMG, MUA, and c‐*fos* showed strong evidence for the focality and steerability of multi‐electrode HD‐ECS. We performed a safety characterization of HD‐ECS for further translational usage. Additionally, the present study confirmed no tissue damage following HD‐ECS, as evidenced by a lack of significant difference in cellular death markers (caspase‐3 and NeuN) and markers for neuroimmune response (Iba1 and GFAP) (Figure 5). Similar brain tissue damage was previously explored in a rat model following ECS^[^ ^32^ ^]^, and no significant damage was observed (as in our case).

### 3.1 Advantages of Using a High‐Density Array

Transcranial electrical stimulation using current pulses has previously been used to evoke motor activity in humans.^[^ ^33^, ^34^, ^35^ ^]^ However, traditional electrode arrangements use centimeter‐scale electrodes in bipolar configurations, which results in diffuse electric fields in the brain and thus stimulation of several regions.^[^ ^36^ ^]^ As a result, off‐target activation resulting in unintended side effects is common. HD electrode arrangements have recently been proposed to enhance focality with tDCS in humans, utilizing a 10–20 montage with electrode distances ≈20% of brain size. Demonstrations with these patterns have been limited to the modulatory effects of tDCS, and the focality of the neural response has not been directly validated. Additionally, the implementations have used static, manually arranged 1 + 4 electrode arrangements^[^ ^35^, ^37^ ^]^, which do not allow dynamic reconfiguration of the injected electric field.^[^ ^25^ ^]^

In this study, we used an HD electrode array scaled to mouse head dimensions, with smaller electrodes (0.4 mm diameter) arranged at a smaller pitch (1 mm). This corresponds to a relative pitch of10% of the mouse head size.^[^ ^25^ ^]^ Therefore, the relative focus is slightly better than that achieved in humans using HD‐tDCS, but the absolute focus of the electric field is an order of magnitude smaller than previously shown. This enables stimulation with high spatial resolution and enhanced focality but requires higher injected current amplitudes to achieve sufficient electric field amplitude in the brain. The array design can be further refined, and higher density electrode arrays can be designed in the future. However, our current electrode sizes and spacings offer a trade‐off between spatial localization and maximum injectable current, limited by the voltage compliance of commercially available multichannel current stimulators. The feasibility of implementing our flexible surface electrode array using a commercially available printed‐circuit board (PCB) manufacturing process is also an important consideration. More flexible arrays can be fabricated using cleanroom techniques, which might improve performance by better conforming to the skull surface, although the cost of development and fabrication would be much higher.

### 3.2 Electronic Control of Electric Field Shape Enables Steerable Stimulation

The use of a dense electrode grid array, rather than individual electrodes or other configurations like concentric ring electrodes^[^ ^25^ ^]^, allows us to electronically control the location of injected currents without having to manually reposition electrodes. Different motor responses were obtained upon changing the electrode pattern (Figure 3), confirming that the electric field did change in the expected manner. Recently, one study found that in a clinical setting, the accuracy of electrode placement on the human scalp was on the order of 6 mm.^[^ ^38^ ^]^ Therefore millimeter‐scale localization of the electric field may not be reliably achievable through manual placement, whereas such movements are easily achieved by electronic control in our method. Scaling up the electrode dimensions, which might be required for human TES, can be achieved without sacrificing precision of steerability by increasing the electrode count and number of stimulation channels in the instrumentation. More complex current‐injection patterns can be obtained via further optimization to allow finer‐grained shifts in the electric field positions (allowing the electric field to be moved at a resolution higher than that of the electrode array).

Steerability is extremely important in practice when stimulation is focal. Individual variations in the site of stimulation^[^ ^22^, ^39^, ^40^ ^]^ require searching for the target during the experiment, which can be done much faster with electronic steerability. Combined with steerability, the high temporal resolution of our stimulation offers another exciting opportunity. Invasively, there is increasing interest in stimulating at multiple locations with high spatiotemporal resolution(e.g. in paired‐pulse stimulation,^[^ ^41^ ^]^ movement disorders,^[^ ^42^, ^43^ ^]^ and stroke recovery^[^ ^42^ ^]^) because, with high spatiotemporal resolution, multisite stimulation can effect changes in neural processing as the processing happens. The steerability and millisecond timescale of our epicranial stimulation can enable similar changes in neural processing noninvasively. Finally, for adaptive implementations not explored in this work, steerability can enable the selection of stimulation patterns based on observed responses (e.g., behavioral or neural).

In addition to changing the position of the stimulated volume, multi‐electrode arrays allow the electric field shape to be adjusted, demonstrated in this paper by adjusting the stimulation's diffusiveness. Other changes to the electric field shape, such as decay rate or field direction, are also possible with more complex electrode configurations. In this work, changing the extent of the stimulation focus in the mouse motor cortex changed the evoked activity between contra‐ and ipsilateral muscles and was consistent with the activation of different ipsilateral pathways. While selectivity in muscle stimulation was shown by preferential activation of certain limbs and whiskers, we still often activated multiple muscles even with HD‐ECS. The benefits of focused electric fields in achieving specific muscle stimulation are likely limited by the overlapping representations of multiple muscles in the motor cortex. This may be improved in larger animals, where representations are more spread out. Additional tuning of injected current waveforms may also improve selectivity by preferentially recruiting certain cell populations and pathways.^[^ ^44^, ^45^ ^]^

### 3.3 From Epicranial to Transcranial

A fully noninvasive transcranial electrical stimulation paradigm implies that the current‐injection electrodes are placed on the surface of the scalp. In this paper, we placed the electrodes directly on the skull to more accurately position the flex‐PCB with respect to potential brain targets. Removing the scalp also allowed us to perform intracortical recording. However, compared to transcranial stimulation through the scalp, we found that injecting the current directly from the skull surface also improved the focus and reduced the required current threshold.^[^ ^31^ ^]^ Further experiments are required to fully characterize the precision and steerability of stimulation with HD patches through the scalp. Nonetheless, the techniques described can also be utilized with chronic subdermal arrays, which would only require a minimally invasive surgery because they would leave the skull intact.^[^ ^31^ ^]^

### 3.4 Clinical and Research Applications of HD‐ECS

The technique used for simultaneous transcranial stimulation and intracortical recording can be extended to gain further insight into the spread of evoked activity across the cortex. A future study using our technology platform could involve discovering whether transcranial stimulation affects the neural tissue in a similar way as the more common intracortical microstimulation (ICMS). Apart from evoking a behavioral response, transcranial electrical stimulation has multiple potential clinical applications, including motor learning enhancement in stroke rehabilitation, behavioral performance enhancement in Alzheimer's patients, modulation of emotional affective neural circuits in depression, and for patients with chronic pain.^[^ ^46^ ^]^ Additionally, HD‐tDCS is known to produce plasticity changes that may outlast conventional tDCS,^[^ ^47^ ^]^ and it has even been shown to reduce the perception of pain in fibromyalgia patients^[^ ^48^ ^]^ and in experimental pain.^[^ ^49^ ^]^ HD‐ECS can be applied to similar applications with improved precision (due to better focusing) and tailoring (due to steerability). Moreover, the electrode array, enabled by fast electronic switching, can also allow near‐simultaneous focused stimulation of multiple sites, which may benefit some brain‐computer interfaces or in the treatment of some neurological disorders like epilepsy.

Many of the clinical applications where intracortical stimulation is useful (e.g., responsive neurostimulation for epilepsy, motor cortex stimulation, somatosensory recovery) can benefit from a less invasive brain stimulation approach such as ECS due to reduced surgical risks and costs. However, because of its low conductivity, the skull is a major barrier to injecting currents into the brain and causes the currents traversing it to disperse. This means that achieving focused stimulation through the skull is a major challenge.^[^ ^31^ ^]^ Our work reduces a layer of surgical penetration, which we believe is a big win in neurosurgical applications.

## 4 Conclusion

This paper demonstrates a novel selective, steerable epicranial brain stimulation technique, which uses HD electrodes to evoke focal responses,validated through behavioral responses (peripheral muscle responses) and intracortical recordings. This rigorous level of validation is unprecedented, both recording local brain activity and correlating it to the evoked behavioral response. The HD design of the electrode arrays enables the use of different electrode combinations to sculpt the electric field of interest within the brain tissue and stimulate different target brain regions, evoking distinct downstream behavioral responses with high spatial resolution. It also enables quick current steering and changes the target by electronically switching the current amplitudes applied to different electrodes, rather thanphysically moving the electrodes, which is a cumbersome process. These novel capabilities (i.e., focal epicranial stimulation evoking behavioral responses and fast electronic steerability ) enable a gamut of new applications, ranging from designing new acute and chronic therapeutic interventions to implementing next‐generation brain‐computer interfaces.

## 5 Experimental Section

### Animal

C57Bl6 mice (6–8‐weeks old) were used in this study. All mice were randomly distributed among different groups. All experiments involving animals were performed in accordance with the Institutional Animal Care and Use Committee guidelines. The use of animals and all procedures were approved by Carnegie Mellon University's Institutional Animal Care and Use Committee (IACUC). Animals were maintained on a 12 h light‐dark cycle with free access to food and water.

### Patch Design and Fabrication

The custom‐designed electrode arrays were purchased from a commercial flexible printed circuit board (flex‐PCB) manufacturing facility (PCBWay, Shenzhen, China), where they were fabricated using standard flex‐PCB technology. Two patch designs were used in this study. The first was a rectangular patch with a 6×4 electrode array in a Cartesian grid layout at a 1 mm pitch, with 0.4 mm‐diameter immersion gold electrodes (Figure 1b). The flex‐PCB stiffness was minimized by using a single‐layer polyimide PCB of nominal 80 µm thickness. The array extent was 5.7 mm×3.7 mm. The signals routed through a monolithic 10 cm‐long flex‐PCB cable to a zero‐insertion‐force (ZIF) connector (FH26 Series, Hirose Electric Co., Tokyo, Japan) that interconnected to a rigid breakout PCB. This patch overlapped the motor cortex in both hemispheres of the mice used in this study. The patch had two screw holes but they were not used to fixate the patch to the skull. Five holes were located between the anterior two rows of electrodes for potential insertion of a recording probe; however, recording probe insertion was not performed with this patch.

The second patch had 27 ring‐shaped electrodes arranged in a hexagonal grid, with 1 mm‐diameter immersion gold electrodes placed at a 1.5 mm pitch. The electrodes had 0.5 mm‐diameter concentric holes, which allowed for insertion of a recording probe (Figure 4a(i)). The electrode area was approximately five times that of the Cartesian 6 × 4 array patch. The electrodes were arranged in six rows that alternated between four and five electrodes across the patch's width. The flex‐PCB, similar to the 6 × 4 array patch, had a nominal 80 µm thickness. The array extent was 7.3 mm × 7.8 mm. The signals routed through a monolithic 15 cm‐long flex‐PCB cable to a ZIF connector, similar to the first patch.

### Surgery for MEP Recording

The animals were deeply anesthetized with a ketamine/xylazine cocktail ((90 mg/10 mg)/kg b.w.) injected intraperitoneally (i.p.), and placed on a stereotaxic apparatus. Local anesthesia, (i.e.,lidocaine (2 mg kg^−1^ b.w.)) was injected subcutaneously (s.c.) at the neck region to reduce pain during surgery. After shaving the mice scalp using hair removal cream, a nick was made with a scalpel, and the scalp was removed to expose the skull. The skull was cleaned with an iodine solution and left to dry before placing the patch. For MEP recordings, Patch 1 (Figure 1b) was placed carefully with reference to the bregma position, targeting the motor cortex of both hemispheres. The patch was held in place with the help of a nylon mesh, and a slow saline drip (2 mL min^−1^) above the mesh was used to improve the electrode‐skull interface and facilitate current injection. For stimulation, the patch was connected to a multichannel stimulation device (micro2+stim, Ripple Neuro, Salt Lake City, UT), and different electrode arrangements (patterns) were delivered through the system using custom code in MATLAB (MathWorks, Cambridge, MA).

### Surgery for MUA Recording

For electrophysiological recordings, following the same surgical procedure mentioned above to expose the skull, a craniotomy was performed by drilling a hole in the right hemisphere of the skull at coordinates AP +1 mm, ML −1.5 mm, targeting the motor cortex. Patch 2 (Figure 4a(i)) was placed on the skull such that the target electrode was aligned with the hole to allow insertion of the multichannel recording probe (H3, Cambridge Neurotech, Newmarket, UK). The probe was inserted up to a DV −1.3 mm depth in the cortex so recording channels covered the whole cortical column.

### Stimulation

Multiple current‐injection configurations, hereafter referred to as electrode patterns, were used to generate different physiological effects. Following the conventions of tDCS electrode montages, patterns were referred to by the number of electrodes used for current injection and return. Hence, 4 + 4 referred to a pattern using eight electrodes in total: four for injection and four for return. Two different patches with two electrode arrangements were used in the present study for different readouts, (i.e., 4 + 4 patterns with Patch 1 for MEP responses and 1 + 3 pattern with Patch 2 for intracortical recordings). Stimulation was performed by simultaneously injecting identical waveforms into all the electrodes (except for sign and amplitude scaling). For MEP and whisker movement, the waveform consisted of trains of seven pulses of 1 ms/phase biphasic pulses repeated at 350 Hz. In every condition, these trains were repeated 10 times at 0.5 Hz. In the intracortical recording experiments, a single monophasic pulse with a 0.2 ms duration was used instead of the pulse train to enable recording of the brain potential at a shorter latency (*t* < 15 ms).

### MEP Data Acquisition

MEP recording was performed in both forelimbs by inserting two 29G stainless steel needle electrodes (DTM‐1.00F, The Electrode Store) in each limb for bipolar measurements. The EMG electrodes were inserted into the triceps brachii muscle of the forelimb. An additional ground electrode was inserted into the base of the tail. The MEP signal was recorded using a bipolar head stage amplifier (RHD 16‐channel bipolar, Intan technologies, Los Angeles, CA) and filtered through a [0.1 Hz; 7.5 kHz] bandpass filter prior to digitization at 20 kHz. To evaluate the relationship between the injected current amplitude and EMG response, an input/output (I/O) curve, was assessed with each pattern. This I/O curve demonstrates the cumulative excitability of large neuronal populations. After acquisition, recordings were analyzed using custom MATLAB scripts. When necessary, the stimulation artifact was first zeroed, and the amplifier recovery from the artifact digitally was digitally compensated by fitting a two‐term exponential following the artifact. The data were then bandpass filtered at [100 Hz; 2000 Hz] using a 10th order Butterworth filter, and an additional 60 Hz notch filter was applied. The peak‐to‐peak amplitude of the response within 100 ms of stimulation was then calculated. Ten trials were applied for each stimulation, and peak‐to‐peak amplitudes were then averaged across trials.

### MUA Data Acquisition

For MUA acquisition, a 64‐channel recording probe (H3, Cambridge Neurotech) was used to record evoked activity following stimulation. The probe was connected to two 32‐channel amplifying head stages (RHD 32‐channel head stage, INTAN technologies). The signal was filtered through a [0.1 Hz; 7.5 kHz] bandpass filter prior to digitization at 20 kHz. After acquisition, the data were analyzed using custom MATLAB scripts. The stimulation artifact was zeroed, then the amplifier recovery from the artifact was compensated by fitting a two‐term exponential following the artifact. The data were then band‐pass filtered at [100 Hz; 2000 Hz] using a 10th order Butterworth filter. High‐impedance channels (*Z* > 1 MΩ) were eliminated from further analysis. The data were interpolated across channels, and a *σ* = 2 Gaussian filter was applied to the image prior to plotting.

### Whisker Movement

A USB camera was used to record whisker movements during stimulation at 30 frames per second. Images were analyzed using custom MATLAB scripts. Two regions of interest surrounding all the whiskers on each side of the face were defined. A global measure of whisker movement amplitude was used by calculating the pixel‐by‐pixel intensity difference between adjacent frames within the six frames surrounding the stimulation. The movement amplitude used was the root‐mean‐square (rms) average (across pixels and time) of the intensity difference in the region of interest.

### Immunostaining

Mice were sacrificed and perfused (cold 4% paraformaldehyde in 1 x PBS) 1 h later to assess cellular and synaptic integrity by labeling for the neuronal marker NeuN (1:500, Abcam), astrocyte marker GFAP (1:500, Abcam), microglia marker Iba1 (1:500, Abcam), apoptosis marker cleaved caspase‐3 (1:200, Abcam), and neural activity marker – c‐*fos* (1:300, Abcam). For immunostaining, 30 µm sections were incubated with blocking buffer (10% normal goat serum and 0.1% Triton X‐100 in PBS) for 1 h. Primary antibodies were diluted in blocking buffer and incubated with the sections overnight at 4 °C. Primary antibodies were visualized using the appropriate secondary antibody conjugates (Alexa Fluor 488, Alexa Fluor 594 ThermoFisher Scientific). The samples were then washed, stained with DAPI (Sigma, #10236276001), and mounted onto glass slides. Images were acquired on a Carl Zeiss Axio Observer microscope using 20× air objectives and subsequently analyzed in ImageJ.

### Statistical Analysis

All statistical analysis was performed using GraphPad Prism Version 9.2. Histology data and behavior data of selective stimulation were analyzed using an unpaired *t*‐test. The steerability data were analyzed by repeated‐measures ANOVA followed by a post‐hoc test with Bonferroni correction for multiple comparisons.

## Conflict of Interest

The authors declare no conflict of interest.

## Author Contributions

V.J. and M.F. contributed equally to this work. M.C. and P.G. developed this idea of non‐invasive brain stimulation. V.J. and M.F. designed and executed the experiments. Analysis using MATLAB scripts and ImageJ software was performed by V.J. and M.F.. C.G. simulated the electric fields for different stimulation patterns. D.Z.T. and G.K.F. designed and fabricated the stimulation patch. V.J. and M.F. wrote the manuscript. All authors discussed and edited the manuscript. M.C. and P.G. supervised the project.

## Data Availability

The data that support the findings of this study are available from the corresponding author upon reasonable request.
